# Euchromatic Transposon Insertions Trigger Production of Novel Pi- and Endo-siRNAs at the Target Sites in the *Drosophila* Germline

**DOI:** 10.1371/journal.pgen.1004138

**Published:** 2014-02-06

**Authors:** Sergey Shpiz, Sergei Ryazansky, Ivan Olovnikov, Yuri Abramov, Alla Kalmykova

**Affiliations:** Institute of Molecular Genetics, Russian Academy of Sciences, Moscow, Russia; Centre National de la Recherche Scientifique Montpellier, France

## Abstract

The control of transposable element (TE) activity in germ cells provides genome integrity over generations. A distinct small RNA–mediated pathway utilizing Piwi-interacting RNAs (piRNAs) suppresses TE expression in gonads of metazoans. In the fly, primary piRNAs derive from so-called piRNA clusters, which are enriched in damaged repeated sequences. These piRNAs launch a cycle of TE and piRNA cluster transcript cleavages resulting in the amplification of piRNA and TE silencing. Using genome-wide comparison of TE insertions and ovarian small RNA libraries from two *Drosophila* strains, we found that individual TEs inserted into euchromatic loci form novel dual-stranded piRNA clusters. Formation of the piRNA-generating loci by active individual TEs provides a more potent silencing response to the TE expansion. Like all piRNA clusters, individual TEs are also capable of triggering the production of endogenous small interfering (endo-si) RNAs. Small RNA production by individual TEs spreads into the flanking genomic regions including coding cellular genes. We show that formation of TE-associated small RNA clusters can down-regulate expression of nearby genes in ovaries. Integration of TEs into the 3′ untranslated region of actively transcribed genes induces piRNA production towards the 3′-end of transcripts, causing the appearance of genic piRNA clusters, a phenomenon that has been reported in different organisms. These data suggest a significant role of TE-associated small RNAs in the evolution of regulatory networks in the germline.

## Author Summary

Silencing of transposable elements (TEs) in germ cells depends on a distinct class of small RNAs, Piwi-interacting RNAs (piRNAs). TE repression is provided by piRNAs derived from large heterochromatic loci enriched in fragmented TE copies, so-called piRNA clusters. According to the current model, individual TEs and their transcripts are considered merely as targets of cluster-derived primary piRNAs, which exert post-transcriptional and transcriptional silencing in *Drosophila*. In our work, we show that natural individual transposons become piRNA-generating loci themselves. We came to this conclusion by comparing the ovarian small RNAs and TE insertion sites of two *Drosophila* strains, which showed that euchromatic target sites of strain-specific TEs generate a number of novel strain-specific piRNAs. This mechanism allows production of additional small RNAs that target active TEs and provide more potent transposon suppression in the germline. Moreover, small RNA production by individual TEs spreads into the flanking genomic regions, which affects the expression of adjacent coding genes and microRNA genes. These data underline the role of individual TEs in a silencing response and explore a new level of TE impact on the gene regulatory networks in the germline.

## Introduction

A large number of transposable element (TE) families populate the genome of *Drosophila melanogaster*, comprising 22% of the whole genome [1]. Four hundred and seventy-eight full-length and 1094 partial-length TE copies were identified in the euchromatic regions of the *D. melanogaster* genome; 436 euchromatic TEs were mapped within transcribed regions, while others were located in intergenic regions [2], [3]. *De novo* TE insertions can result in alterations of gene expression. TEs are considered to be co-evolving with host genomes, providing cellular genes with new regulatory signals [4]. The majority (77%) of the sequenced heterochromatin in *D. melanogaster* is composed of TEs [5]. Most of the heterochromatic TEs are destroyed by insertions of other transposons, are not capable of autonomous transposition, and are concentrated in a number of mainly pericentromeric regions. These regions—termed piRNA clusters—were previously considered ‘junk’ DNA and are implicated in a defense system called the Piwi-interacting RNA (piRNA) pathway [6], [7]. Heterochromatic transposon-dense loci produce abundant piRNAs that regulate transposon activity in gonads [6]. Primary piRNAs are processed from putative long, single-stranded transcripts encoded by piRNA clusters and demonstrate a strong 5′ terminal uridine bias (1 U bias). In germ cells, most piRNA clusters contain a mixture of sense- and antisense-oriented TE fragments, are transcribed from both strands and therefore generate piRNAs of both polarities [6]. In the follicle (somatic) cells, single-stranded clusters (e.g., X-linked *flamenco* locus) contain transposon fragments inverted relative to the direction of transcription and produce piRNAs that are almost exclusively antisense to TEs [6], [8]. In germ cells, secondary piRNAs are generated through piRNA-guided cleavage of transposon mRNA, so that primary antisense piRNA and newly produced sense piRNA have a 10-nt complementarity overlap at their 5′-ends [6], [8], [9]. Active expression of transposable elements provokes amplification of cluster-derived antisense piRNAs (the ‘ping-pong’ mechanism), reinforcing the suppression of TE activity in the germline. piRNAs mediate post-transcriptional TE silencing through the homology-dependent cleavage of the cognate transcripts [7], [9], [10]. Additionally, piRNAs may exert *transcriptional* silencing of TEs in the *Drosophila* ovaries [11], [12], [13]. According to the original ‘ping-pong’ model, mRNAs of the euchromatic TEs are considered as targets of cluster-derived antisense piRNAs [6]. A notable exception is the retrotransposon *Ulysses* in *D. virilis*, which produces 99% of primary piRNAs corresponding to its coding strand [14]. In mice testes, individual TE coding transcripts are recognized as a source of primary piRNAs [15]. piRNA clusters also produce significant amounts of endogenous small interfering RNAs (endo-siRNAs) in a Dicer-2-dependent manner [16], [17], [18]. Thus, both pi- and endo-siRNA pathways silence transposons in ovaries.

Recently, we showed that transgenic constructs containing a transcribed fragment of *Drosophila* transposon *I*-element become *de novo* piRNA and endo-siRNA producing clusters that are reminiscent of native dual-stranded clusters [19]. Small RNAs of both polarities were shown to be generated from the entire transgene and flanking genomic sequences. It was proposed that transgenic transcripts can be recognized by Piwi loaded with endogenous *I*-specific piRNAs. This event leads to site-specific chromatin modifications followed by processing of the transgenic transcripts into piRNAs. Here, we extend this finding by showing that in the germline, euchromatic copies of TEs also become a source of additional piRNAs. piRNAs are generated not only from the transposons themselves but also from the flanking genomic sequences. This integration-dependent piRNA cluster formation is observed for all classes of TEs and represents a mechanism that adds a new layer to piRNA-mediated protection against TEs. We speculate that the piRNA pathway might cooperate with the endo-siRNA response to silence novel TE integrations in the germline. Our data underline the role of individual TEs in the defense against transposon expansion and explore a new level of TE impact on gene expression in the germline.

## Results

### Insertion of TEs into euchromatin induces small RNA production at their target sites

Given that transgenes containing transcribed retrotransposon fragments form *de novo* piRNA clusters [19], we asked whether insertions of natural TEs into euchromatic regions can also induce piRNA cluster formation. Since the repetitive nature of TEs impedes mapping of small RNAs to particular TE copies, we focused on testing the possibility that newly inserted TEs are capable of inducing small RNA generation from adjacent genomic regions that normally do not produce small RNAs. Analysis of individual TE insertions within one strain would not allow discrimination between pre-existing and *de novo* TE-induced piRNA clusters; therefore, to systematically test this hypothesis, we compared genomes and ovarian small RNAs from isogenic *y^1^; cn^1^ bw^1^ sp^1^* and the *w^K^ D. melanogaster* strains. The *y; cn bw sp* strain was previously used for whole-genome sequencing by the Drosophila Genome Project, while the ovarian small RNA library of *w^K^* was reported previously [19]. In addition, we performed deep sequencing of small RNAs from ovaries of *y; cn bw sp* (GSE46105) and whole genome sequencing of the *w^K^* strain (SRR831712).

Based on the well-defined genome annotation of the *y; cn bw sp* strain, we identified a set of 463 full-length and 2622 partial (>180 bp) individual euchromatic TEs located outside of known piRNA master loci [6] (Materials and Methods). We identified 348 full-length and 302 partial TEs that are specific for *y; cn bw sp* and absent from the *w^K^* genome by applying the deletion search tool [20] to the assembled *w^K^* genome. To validate the deletion search procedure, we confirmed the absence of several TEs in *w^K^* by PCR (Table S1).

To exclude the possibility that TE insertions specific for the *y; cn bw sp* strain occurred in preexisting small RNA-producing regions, we checked whether or not insertion sites of these TEs generate small RNAs in the *w^K^* strain. For this, we applied a stringent requirement that small RNA reads should be absent in the *w^K^* strain and ≥1 reads should be present in the *y; cn bw sp* strain within a narrow region adjacent to TEs (100 nt). This is necessary to avoid counting small RNAs that can occasionally be produced by nearby transcribed regions. This procedure allowed us to identify 185 full-length and 106 partial-length TEs that are specific to the *y; cn bw sp* strain and co-localize with regions producing small RNAs unique to this strain. In total, 5502 and 3177 reads for the full-length and fragmented TEs, respectively, originating from both sides of the 1-kb regions flanking TEs, were identified. The same genomic regions lacking TE insertions in the *w^K^* strain produced only 517 small RNA reads. For further analysis, we used a set of 91 full-length and 30 partial TEs (‘master list’, Table S1) that had at least five small RNA reads within the 1-kb flanking regions from both sides of the TE insertions.

Small RNAs derived from the 1 kb regions flanking all euchromatic TEs (>180 nt) within the genome of *y; cn bw sp* strain is ∼10 times less abundant than unique small RNAs originating from the annotated heterochromatic piRNA clusters [6] (35494 and 369739 reads, respectively).

Next, we performed a similar analysis for the *w^K^* strain. Using a recently described approach for unassembled genomes [21] (Materials and Methods), we identified 1089 euchromatic insertions of TEs that are specific to *w^K^* and absent from the *y; cn bw sp* genome. At least 96 of these insertions are located within regions producing abundant small RNAs specific to the *w^K^* strain (8797 reads within 1-kb TE flanking sequences) (Table S2). These findings suggest that the insertion of TEs into euchromatic loci induces the generation of novel small RNAs from these regions.

### Characteristic features of small RNAs associated with euchromatic TE insertions

**Figure 1:**
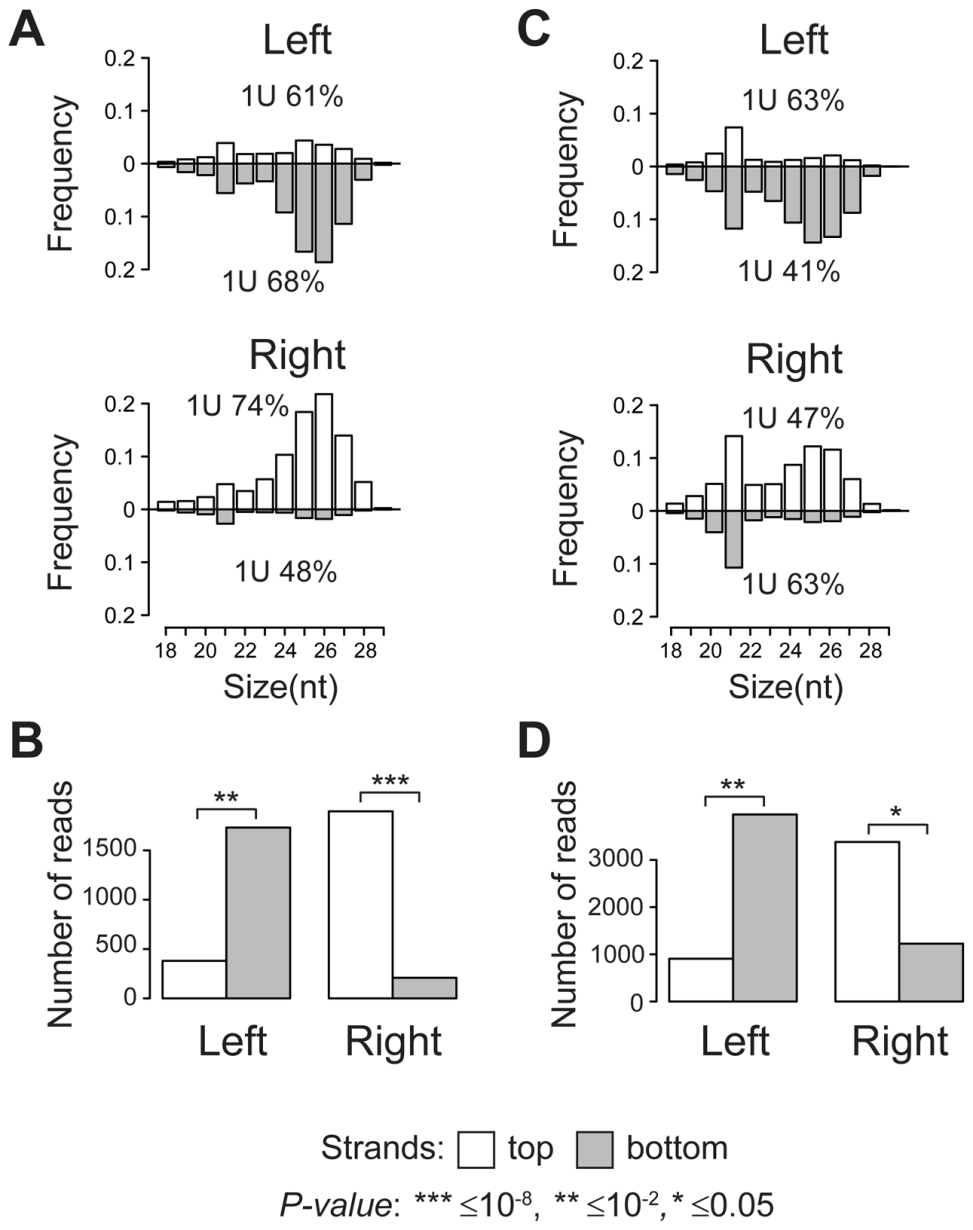
TE insertions induce asymmetric piRNA production in the adjacent genomic regions. Length distribution of small RNAs mapped to the TE flanking regions in the *y; cn bw sp* ((A), full-length TEs from Table S1) and *w^K^* ((C), TEs from Table S2) strains. Percentage of reads having 1 U is indicated for each strand. *P-value* was evaluated by two-sided Student's t-test. The amounts of small RNAs produced by the opposite strands of the TE flanking sequences in the genome of *y; cn bw sp* ((B), full-length TEs from Table S1 are considered) and *w^K^* ((D), TEs indicated in Table S2 are considered).

Size distribution analysis of TE-associated small RNAs showed that the majority of small RNAs produced at TE target sites are 24–29 nt in length with a strong 1 U-bias, which is a characteristic of piRNAs (Figure 1A, C). A clear peak at 21 nt indicates that endo-siRNAs are also generated. Surprisingly, a strong strand asymmetry in small RNA production relative to TE was observed. Independent of the TE orientation in the genome, the left flanks generate predominantly small RNAs from the negative genomic strand, while the majority of the small RNAs from the right flanks are mapped to the positive strand (Figure 1B, D; Table S1, S2). Moreover, we observed a number of 24–29 nt piRNAs that overlapped the borders between full-length TEs specific to *y; cn bw sp* strain and their neighboring genomic sequences. These reads were also asymmetrically distributed (Figure S1, A, B, C). These observations imply that piRNA precursor transcripts of both polarities are most likely initiated within TE and extend into neighboring genomic regions. This transcriptional mode may be defined as divergent. Using strand-specific RT-PCR analysis, we confirmed that *blood* insertion in the intergenic region induced divergent transcription from TE into flanking sequences (Figure S2).

Eleven per cent of full-length TE insertions common to both strains were located within regions producing small RNAs (Figure S3, Table S3). For these copies, we observed the same asymmetric mode of small RNA distribution as for the strain-specific TEs. Thus, euchromatic TE insertions induce formation of the asymmetric piRNA clusters.

The predominance of the intact full-length TE copies in the master list (76%; Table S1) and the absence of a strong preference to any TE class (*P-value*>0.01, Chi-square test) supports the idea that the active transcription of TEs is a prerequisite for piRNA cluster formation at TE integration sites. The fact that certain partial TEs also induce piRNA production in an asymmetric mode (e.g., *Stalker* solo-LTR (long terminal repeat), Table S1), suggests that these TE fragments contain active promoters. Twenty-six per cent of the strain-specific full-length TEs (91 from 348 TEs) are capable of inducing potent production of the piRNAs at the sites of integrations. We cannot exclude that the efficiency of the TE-associated piRNA clusters also depends on the transcriptional and chromatin status of the target region. The lower rate of piRNA cluster formation at individual full-length TE integration sites common to both strains can likely be explained by their older evolutionary age and therefore transcriptional inactivity.

**Figure 2:**
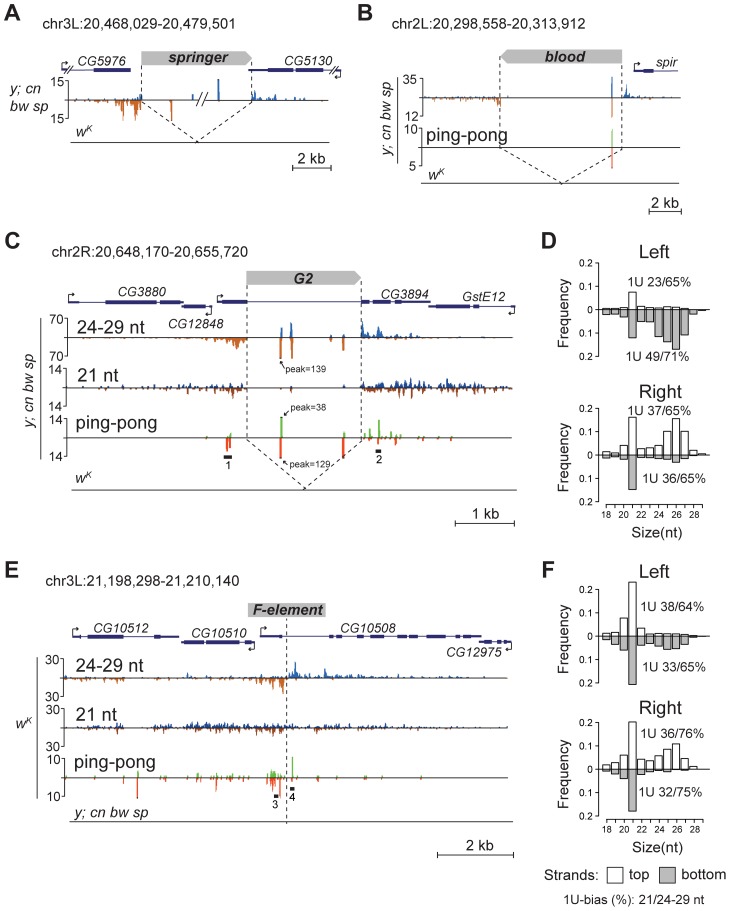
TE insertions generate double-stranded mixed pi- and endo-siRNA clusters in the germline. Small RNA density (number of reads; no mismatches allowed) at the selected genomic loci containing TE insertions unique for the *y; cn bw sp* (A, B, C) or *w^K^* (E) strains. The coordinates of the depicted genomic regions are indicated. Length distribution of small RNAs mapped to the *G2* flanking regions in the *y; cn bw sp* (D) and *F*-element in *w^K^* (F). Percentage of 21-nt and 24–29-nt reads having 1 U is indicated for each strand. Broken arrows indicate the direction of gene transcription. Small RNA plots for *w^K^* (A, B, C) and *y; cn bw sp* (E) are presented at the same scale as corresponding plots for *y; cn bw sp* and *w^K^*, respectively. Reads mapped to the sense strand are shown in blue, and antisense in brown. Ping-pong pairs are shown in green-red. Ping-pong pairs corresponding to regions 1, 2 (C), and 3, 4 (E) are shown in Figure S4.

To confirm that TEs themselves also generate piRNAs, we inspected some euchromatic copies that have unique small RNA reads mapping to them. We found that such TEs indeed produce piRNAs of both polarities (Figure 2A, B, C). Together, these data suggest that, when located in euchromatic regions, active TEs form piRNA clusters and induce production of novel piRNAs from the flanking genomic regions.

We noticed that small RNA production induced by some TEs unique to either *y; cn bw sp* or *w^K^* could spread at distances up to several kilobases away from the integration site (Figure 2C, E). These small RNAs are represented by piRNAs showing a strong 1 U-bias and by 21-nt RNAs with a moderate 1 U-bias (Figure 2 D, F). 1 U small RNAs show a 10-nt 5′ overlap with small RNAs mapping to the opposite strands, which is a signature of the ping-pong cycle (Figure 2C, E). We found mixed ping-pong pairs of pi- and siRNAs (Figure S4). Interestingly, potent endo-siRNA production spreads across a long range, while piRNAs are mostly located close to TE ends and their abundance decreases rapidly with distance from integration sites. Possibly, the endo-si- and piRNA pathways can cooperate to amplify small RNAs from the TE insertion sites in the *Drosophila* germline.

### Prediction of TE insertions based on the piRNA profile

**Figure 3:**
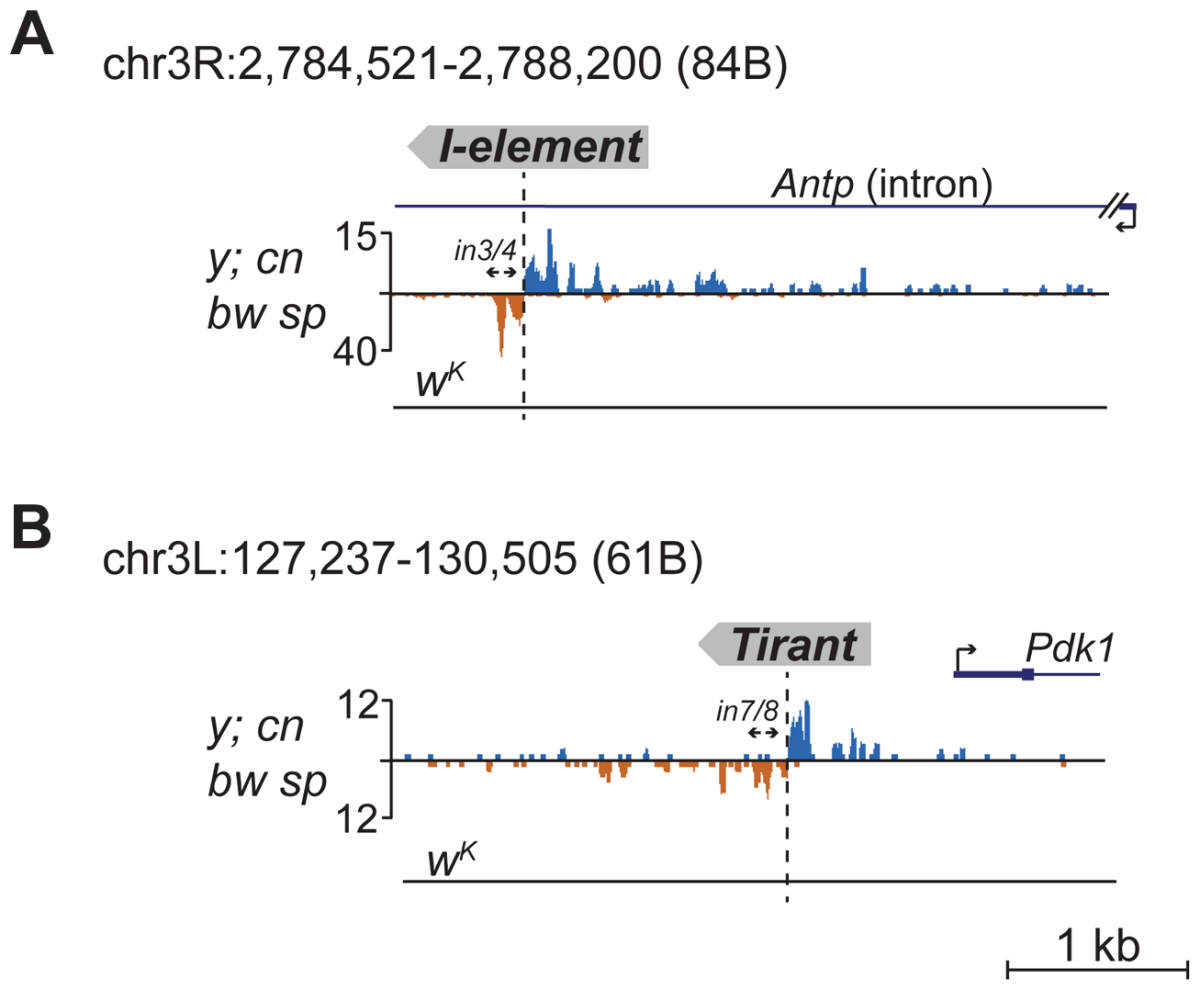
Detection of newly transposed TEs in the *y; cn bw sp* genome based on the piRNA profile. Diverged piRNA clusters were detected within 84B (A) and 61B (B) loci lacking annotated TEs. Inverse PCR was done using the primers indicated by arrows. Sequencing revealed an *I*-element insertion in 84B (A) and *Tirant* in 61B (B). The coordinates of the depicted genomic regions are indicated.

Detection of distinctive divergent piRNA clusters associated with TE insertions suggests that the presence of such clusters may itself serve as an indicator of newly transposed TEs. To confirm this, we searched for divergent piRNA clusters within *y; cn bw sp* genomic loci that did not correspond to annotated TEs. Such clusters were indeed detected (Table S4). To recover some of these putative insertions, we performed inverse-PCR using genomic DNA of the *y; cn bw sp* strain as a template. Sequencing of the inverse PCR products revealed an *I*-element within the 84B locus (Figure 3A), *Tirant* within loci 45F and 61B (Figure 3B, Table S4), *Doc* within 84B, and *F*-element within 89B (Table S4). Strikingly, we detected TEs in the center of asymmetric piRNA clusters in five cases out of five analyzed, which confirms that formation of asymmetric piRNA clusters at the sites of recent TE insertions is a general phenomenon.

Many of the LINE (long interspersed nuclear element) retrotransposons are present in the master list of TEs that induce piRNA production from the flanking regions. However, *I*-element-related piRNA clusters were not detected in the analysis (Table S1). This was particularly surprising, because transgenes containing fragments of the *I*-element are capable of forming piRNA clusters, as previously reported [19]. *I*-elements were shown to be highly unstable and polymorphic in the genome of the *y; cn bw sp* strain used for sequencing and annotation of the *Drosophila* genome [22]. Therefore, differences in the *I*-element insertion sites between the reference genome and the genome used for small RNA sequencing might explain this discrepancy. Here, we show that natural *I*-element insertions are also able to induce piRNA cluster formation in euchromatin (Figure 3A). For small RNA sequencing, we used a sub-line of *y; cn bw sp* maintained in our laboratory for 15 years. We analyzed the genomic distribution of *I*-elements in our *y; cn bw sp* sub-line by FISH on polytene chromosomes and found significant differences compared to annotated *I*-element sites (Table S5). Of note, the *I*-element was indeed detected within the 84B locus. We can therefore conclude that asymmetric profiles of piRNA density in euchromatin may be used for the prediction of *de novo* TE insertions.

### Individual TEs trigger the production of piRNAs from adjacent cellular genes

**Figure 4:**
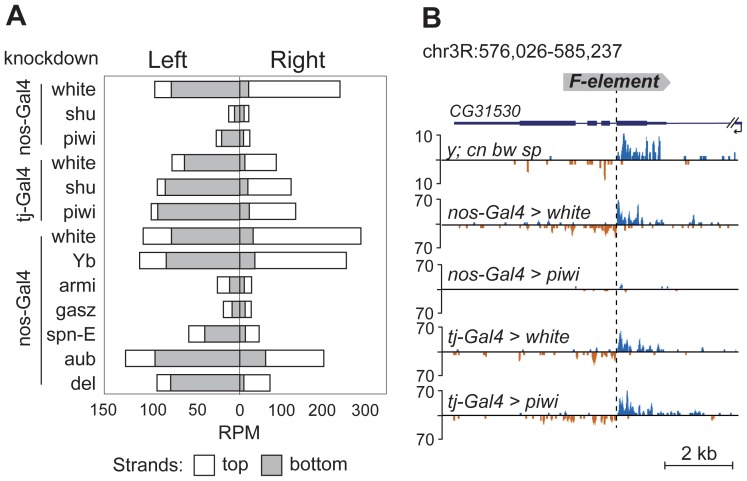
TE-flanking regions produce genuine piRNAs. Analysis of small RNA data from piRNA pathway gene knockdowns [24], [25]. (A) Bar graphs represent normalized numbers of small RNAs (in reads per million, rpm; no mismatches allowed) corresponding to the left or right flanks of TEs in different knockdowns. Only TEs common to *y; cn bw sp* and strains used for knockdown experiments were analyzed. Small RNAs mapping to the positive (white) or negative (grey) strands are shown. (B) An effect of *piwi* knockdown in germ and somatic cells on small RNA abundance in the vicinity of *F*-element insertion within *CG31530* gene. The coordinates of the depicted genomic region are indicated.

TEs were previously grouped into germline-specific, somatic, and intermediate groups based on the changes of steady-state RNA levels and piRNA content in the ovaries of piRNA pathway mutants [8], [23]. Suppression of TE expression in germ cells depends on Ago3 and Aub proteins that are involved in a ping-pong piRNA amplification loop. Somatic TEs are transcriptionally active in follicular cells and their silencing is not dependent on Ago3 or Aub. TEs from the intermediate group are active in both cell types. Approximately 80% of the TE master list consists of germline-specific TEs and ∼20% of intermediate TEs (Table S1), indicating that TE-associated piRNA cluster formation occurs in the ovarian germ cells. To confirm this, we analyzed small RNA libraries prepared from somatic and germline knockdowns of the piRNA pathway genes. Specifically, we used ovarian small RNA libraries from strains with *nanos*- or *traffic jam*-driven knockdown of *white*, *piwi*, or *shutdown* [24] and *nanos*-driven knockdown of *white*, *Yb*, *armitage*, *gasz*, *spindle-E*, *aubergine*, and *deadlock* [25] (*nanos* promoter is germline-specific, *traffic jam* promoter is follicular cell-specific; Figure 4). We found that the flanking regions of 12 TEs from our master-list (Table S1) also generate significant amounts of small RNAs in strains used in knockdown experiments indicating the presence of these TE insertions in these strains. We observed a drastic decrease in the TE-flanking read content upon germline-specific knockdown of Piwi, Armitage, Spindle-E, Shutdown and GASZ (Figure 4). Thus, euchromatic TE insertions induce formation of genuine piRNA clusters in the germline.

**Figure 5:**
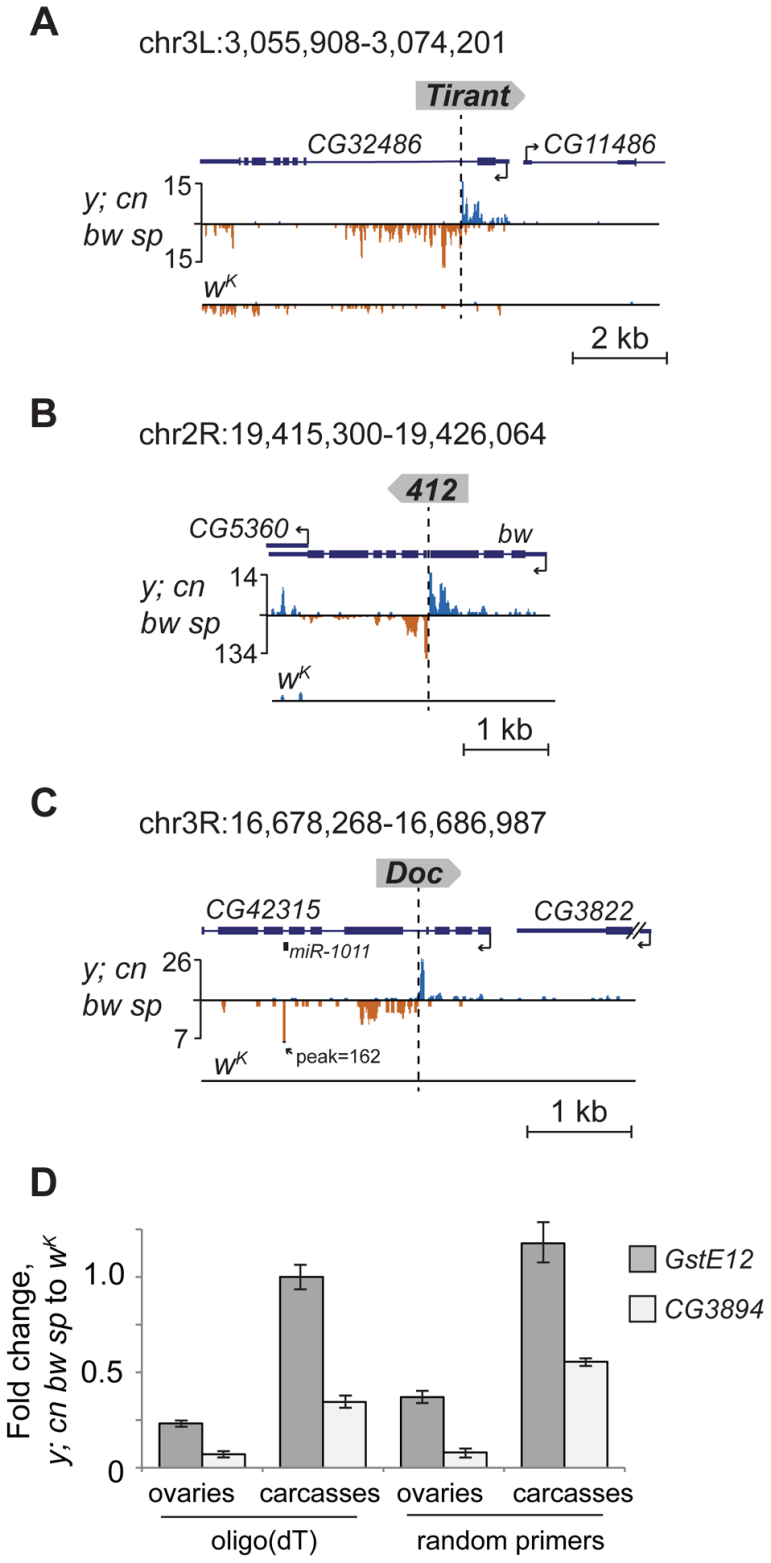
TE insertions induce piRNA production from the nearby genes. Small RNA density (number of reads; no mismatches allowed) at the genomic loci containing TE insertions unique for the *y; cn bw sp*. The coordinates of the depicted genomic regions are indicated. TE insertions induce the generation of antisense relative to gene piRNAs from the neighboring gene exons (A, B). (C) Insertion of the retrotransposon *Doc* in the intron of *CG42315* induces production of *miR1011* located within the intron of this gene. (D) RT-qPCR analysis of the amount of *CG3894* and *GstE12* gene transcripts in ovaries and carcasses of *y; cn bw sp* and *w^K^* strains (genomic region is shown in Figure 2C). Histogram bars represent a normalized ratio of gene transcript abundance in the ovaries of *y; cn bw sp* to that of *w^K^* strain. Reverse transcription was done using oligo(dT) or random primers. Error bars indicate standard deviation of triplicate PCR measurements for two independent RNA samples.

Appearance of TE integration-induced piRNAs may exert a considerable effect on the expression of nearby genes in ovaries. To address this, we analyzed piRNA clusters generated by individual TEs in different genomic surroundings. Forty-seven per cent of TEs were located in the intergenic regions, 47% within introns, and only four insertions overlapped with exons (Table S1). Some intergenic and intronic TE copies formed piRNA clusters that spread into neighboring genes. We found that some TEs inserted into introns had induced the generation of antisense small RNAs relative to neighboring exons (Figure 2A, C, E; Figure 5A, B, C). TE insertion within or near genes could affect gene expression by different means including disruption of transcription units, promoter replacement, or competition. In these cases, changes in gene expression may be considered as a direct consequence of insertion leading to structural alterations. We compared expression of *CG3894* and *GstE12* genes located within a mixed pi- and siRNA cluster induced by the insertion of *G2* element in *y; cn bw sp* (Figure 2C) to their expression in *w^K^*. In order to determine the effect of insertion *per se*, this experiment was performed using RNA extracted separately from ovaries and carcasses. We observed a strong repression of *CG3894* and *GstE12* expression in ovaries of the *y; cn bw sp* strain (Figure 5D). piRNAs may also be expected to modulate the expression of genes *CG5976*, *CG5130* (Figure 2A), and *CG32486* (Figure 5A). However, these genes did not show significant difference in expression level in *y; cn bw sp* where TE insertions are detected compared to *w^K^* (not shown).

Insertion of the retrotransposon *Doc* in the intron of *CG42315* represents another example of the impact of TE-associated piRNA cluster formation on the host genome (Figure 5C). In this case, TE-induced small RNA production spreads into the mirtron that encodes the somatic microRNA *dme-miRNA-1011* (miRBase data), which resulted in ectopic miRNA overexpression within ovaries where this miRNA is normally not expressed.

### TE insertions in the 3′ untranslated region (UTR) of actively transcribed genes induce piRNA production towards the 3′ end of gene transcripts

**Figure 6:**
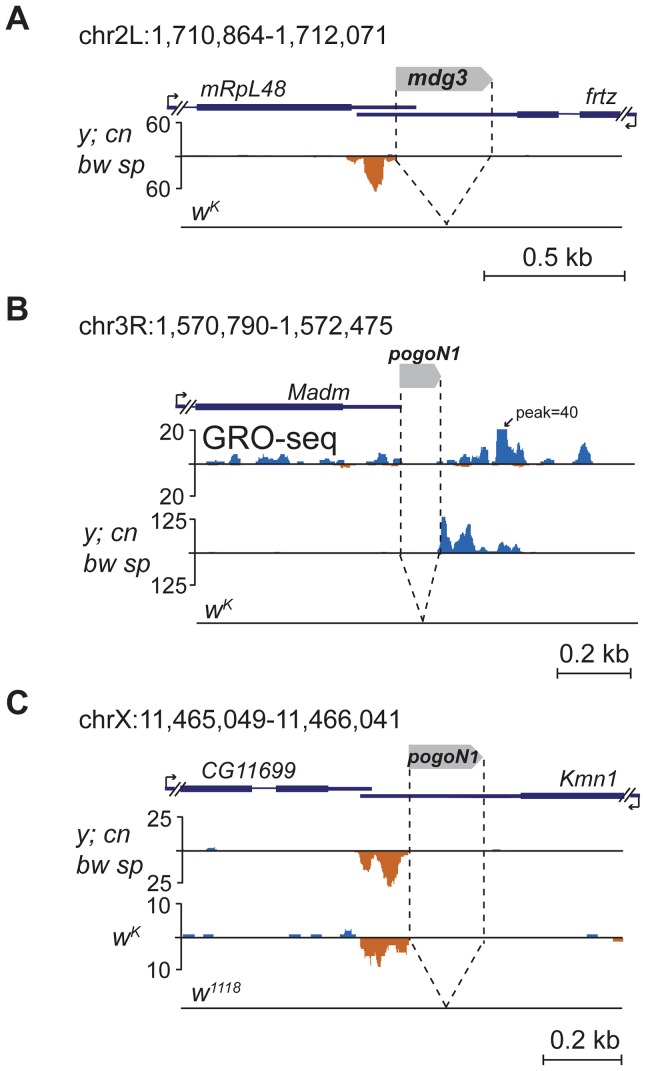
TE insertions into the 3′UTR regions induce formation of the 3′-directed single-stranded piRNA clusters. Small RNA density (number of reads; no mismatches allowed) at the genomic loci containing TE insertions into the 3′UTRs. The coordinates of the represented genomic loci are indicated. (A) Insertion of *mdg3* solo-LTR. (B) Insertion of *pogoN1* unique for the *y; cn bw sp* strain. GRO-seq density (data from [12]) is displayed to show that the *pogoN1* insertion is located within the 3′ region of the *Madm* gene transcript. (C) Insertion of *pogoN1* in the 3′UTR of the *kmn1* gene revealed in *y; cn bw sp* and *w^K^* strains is absent in the *w^1118^* strain.

Several TE insertions mapping to the 3′ non-coding exons induce single-stranded asymmetric piRNA production downstream of insertion sites towards the 3′ end of the gene. Such an arrangement is characteristic of insertions of short TEs or TE fragments into the 3′UTR of genes actively expressed in the ovaries. For example, insertion of *mdg3* solo-LTR in the 3′UTR of non-piRNA producing gene *ftrz* stimulates piRNA production in *y; cn bw sp* strain (Figure 6A). Several events of piRNA cluster formation within 3′UTRs were associated with insertions of non-autonomous transposon *pogoN1* (Table S6). We found polymorphic *pogoN1* insertions specific for the *y; cn bw sp* strain, which provokes the formation of unidirectional piRNA clusters (Table S6). Despite the fact that some of these insertions were annotated downstream of genes, analysis of the ovarian global run-on (GRO)-seq [12] clearly shows that these insertions are located within the 3′ region of neighboring gene transcripts; for example, *pogoN1* insertion close to the *Madm* gene (Figure 6B; Table S6). *pogoN1* induces piRNA production towards the 3′ end of gene transcripts independently of its orientation (Figure 6B, C; Table S6). We also found that the *pogoN1*-associated piRNA cluster detected in the 3′UTR of the *kmn1* gene in *y; cn bw sp* and *w^K^* strains (Figure 6C) was absent in strain *w^1118^* (data from GSM919410, GEO) which lacks this insertion (Figure 6C). piRNAs complementary to *pogoN1*, both sense and antisense, are present in the total piRNA population of *y; cn bw sp* strain, which indicates their germline origin from dual-strand piRNA clusters. 5′ RACE showed that the only detected transcription start-site of the fused *kmn1-pogoN1* transcript coincides with *kmn1* mRNA 5′ end indicating that the transposon is lacking its own promoter activity and is transcribed as part of a gene transcript (not shown). This conclusion is confirmed by the fact that the majority of *pogoN1* copies located outside of genes do not induce piRNA production (Table S6). According to our hypothesis, the fused transcript containing transposon sequences within its 3′UTR is recognized by sense or antisense TE-specific piRNAs (depending on the TE orientation) and processed into piRNAs only in one direction, towards the 3′ end, suggesting that only the 3′ RNA product of the endonucleolytic cleavage of the piRNA-Piwi protein complex is involved in further piRNA processing. Intronic *pogoN1* insertions do not induce piRNA cluster formation (Table S6), indicating that primary piRNA processing occurs downstream of splicing.

## Discussion

**Figure 7:**
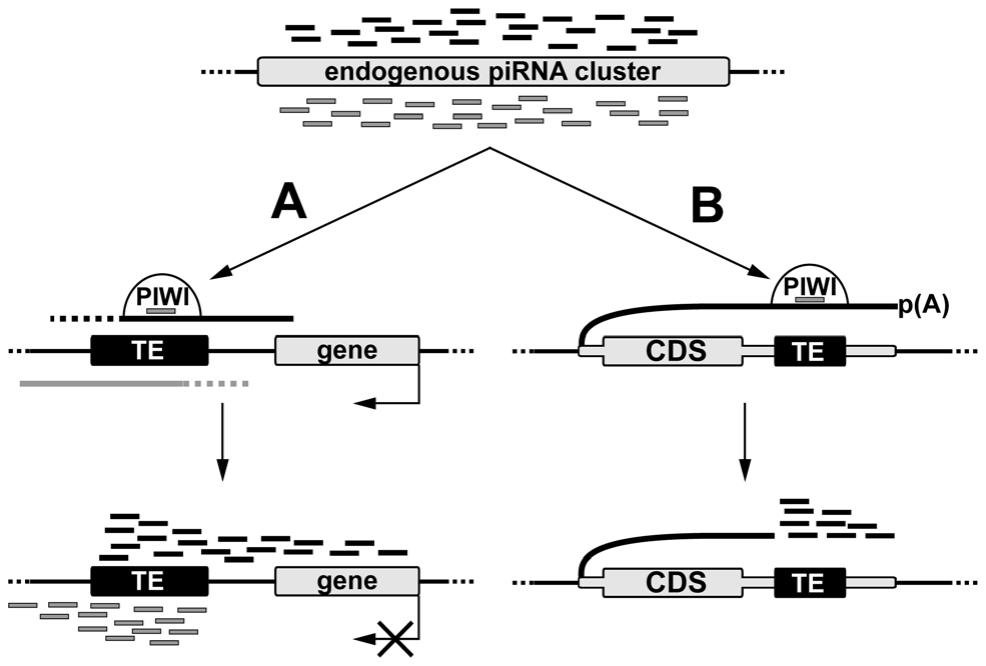
A model of *de novo* piRNA cluster establishment at euchromatic copies of TEs. piRNAs produced by endogenous clusters and loaded in the nuclear Piwi protein recognize transcripts containing TE sequences and initiate processing of these transcripts into piRNAs. (A) In case of TE integration into intergenic region small RNA production spreads into the flanking genomic regions in an asymmetric fashion. Nature of promoters driving sense and antisense transcription is currently unknown. Formation of the TE-associated piRNA clusters can affect expression of the nearby cellular genes. (B) Integration of TE in the 3′UTR of a cellular gene causes piRNA production towards the 3′ end of the chimeric TE-containing transcript.

Comparative genome-wide analysis of TE insertions in two *Drosophila* strains allowed us to discover that recently transposed euchromatic TEs become active piRNA clusters that are associated with piRNA production from the TE flanking regions (Figure 7). Production of small RNAs by individual TE copies in *Drosophila* is reminiscent of processes described for the mammalian germline where the main source of piRNAs at pre-pachytene stages is individual TE copies rather than piRNA clusters [15]. Evolutionary conservation underlines the significance of piRNA production by individual TEs. TE-associated piRNA clusters in *Drosophila* may be defined as divergent, similar to pachytene piRNA clusters previously described in the mouse. Similar asymmetric profiles of the piRNA distribution were found in the vicinity of the *Caenorhabditis elegans* TEs [26]. Divergent murine piRNA clusters are transcribed from the shared bidirectional promoter located between the two transcription start-sites [27].

TE-dependent piRNA cluster formation requires the existence of both sense and antisense transcription at the site of TE integration. While our data indicate that the promoter activity of a particular TE copy is a prerequisite for piRNA cluster formation, the origin of antisense transcripts is not always obvious. There are three possible sources of sense and antisense transcripts that give rise to piRNAs at *de novo* TE integration sites in *Drosophila*. First, divergent transcription can be initiated at discrete sense and antisense promoters within TEs and continue into neighboring genomic regions, thus providing precursor transcripts that are recognized by the endogenous TE-specific piRNAs and are further processed into additional piRNAs. Indeed, bidirectional transcription from the closely located promoters was reported for the human LINE1 and a few *Drosophila* non-LTR retrotransposons [28], [29], [30], [31]. However, our data suggest that TEs from almost all families analyzed are able to produce transcripts of both polarities extending beyond the TE, which indicates that all TE classes should possess both sense and antisense promoters. However this requires further investigation. The second possibility is that pervasive transcription of the genome, including loci containing *de novo* TE insertions, is the source of RNA for piRNA processing. However, it is not always the case, since the antisense transcription of a particular locus (Figure 2B) was strongly stimulated by *blood* integration (Figure S2). Finally, it is possible that bidirectional transcription of TE-dependent piRNA clusters is a result of piRNA activity. Previously, we have shown that transgenes initially comprising two genes driven by co-oriented *hsp70* promoters become *dual-stranded* piRNA clusters [19] suggesting that piRNA cluster formation and initiation of bidirectional transcription may be interdependent processes. It is possible that epigenetic modifications accompanying piRNA cluster formation stimulate bidirectional transcription of the locus by an unknown mechanism followed by processing of these transcripts into piRNAs. It was observed that not all TE copies (this paper) or transgene insertions [19] induce potent piRNA clusters suggesting that *de novo* piRNA cluster formation depends on the transcriptional and chromatin status of the target region. Previously, it was shown that certain chromatin domains prevent Piwi-mediated chromatin spreading [32]. We speculate that all three scenarios contribute to *de novo* dual-stranded piRNA cluster formation within different genomic contexts for different classes of TEs.

An important prediction of our model is that non-annotated TE insertions may be detected by the presence of diverged piRNA clusters. Based on the presence of such clusters, we found newly transposed TEs in the loci lacking annotated TEs. Using this approach, we have found insertions of the *I*-element, which is unstable in the genome of the reference strain *y; cn bw; sp* and, as a consequence, has a sub-line-specific pattern of localization. Thus, divergent piRNA clusters may be used as a tool for detecting polymorphic TE insertions in non-annotated genomes.

In *C. elegans*, piRNAs initiate the production of secondary endo-siRNAs at sites that are complementary to piRNAs [26]. We have found that in some cases, *Drosophila* TE insertions also induce the production of endo-siRNAs of both polarities spreading far into the adjacent regions. Previously, we have shown that transgenes containing the *I*-retrotransposon fragment induce production of pi- and siRNAs from the nearby genomic sequences [19]. Pronounced endo-siRNA clusters in close proximity to TEs were found only in a few cases implying that a specific genomic context is required for their formation. Most likely, active transcription units located in proximity to TE insertions provoke endo-siRNA generation by providing precursor transcripts for their production. Endogenous major piRNA clusters also produce abundant endo-siRNAs in the germline, acting alongside the piRNA pathway [16], [17], [18]. In our recent study, we have shown that transgenes containing transposon fragment produce 21-nt RNAs and piRNAs that are capable of forming ping-pong pairs [19]. Heterologous pi-siRNA ping-pong pairs were revealed in the TE flanking regions (Figure S4). Possibly, the repeat-associated endo-siRNA pathway cooperates with the piRNA pathway in the germline to amplify small RNAs from the TE insertion sites.

It was recently reported that piRNA-mediated heterochromatin spreading affects the expression of the genes located close to TE insertions in *Drosophila* ovarian somatic cells [32]. Here, we have shown that piRNA production triggered by the TE insertions spreads into TE flanking genomic regions in the ovaries. Because of the strong decrease in the abundance of piRNAs coming from the TE flanking regions upon Piwi knockdown in the germline but not in the follicular cells, we suggest that this phenomenon occurs in the germline. The main purpose of small RNA generation and chromatin modifications is to silence TEs. The production of piRNAs from the TE-flanking regions seems to be a side effect of the *de novo* cluster formation. However, our data indicate that these small RNAs can change gene expression in the germline. Taking into account that TE-flanking piRNA population is associated with recent TE transpositions, its content may be considered as strain- or individual-specific, which could provide intraspecies variability of gene expression in the germline. Even in the cases of intergenic or intronic TE locations, spreading of small RNA production into exons can result in the appearance of gene-specific piRNAs, which could affect target gene expression in the germline. Moreover, chromatin changes accompanying piRNA cluster formation could diminish target gene accessibility to RNA pol II in the germ cells. These data suggest that intronic or intergenic TE insertions may be evolutionarily significant and be a subject to natural selection.

TE insertions into the 3′UTR regions are of particular interest due to their ability to induce the formation of 3′-directed single stranded piRNA clusters, which has been reported for other organisms [33], [34], [35]. It was previously observed that 3′UTRs of some mRNAs are processed into piRNAs in *Drosophila*, murine, and *Xenopus* gonads [34], [35]. The role of the 3′UTR-derived piRNAs remains unclear. Previous studies noted that the murine *Tcfcp2l* gene has numerous repetitive elements resident in its 3′UTR, suggesting their potential relationship to the piRNA production from the 3′UTR [33]. However, in this case, no direct correlation between TE insertions and piRNA production was established. We have shown that solo-LTR and transposon insertions in the 3′UTR of genes that are expressed in ovaries, but which normally do not produce piRNAs, induce sense-piRNA production starting from the insertion site towards the 3′ end of the transcript. We propose that endogenous piRNAs recognize gene transcripts comprising a transposon fragment, causing their endonucleolytic cleavage, which stimulates further processing of the transcript into piRNAs only in one direction, towards the 3′ end (Figure 7). Most likely, the 5′-monophosphate terminus resulting from the enzymatic activity of Piwi proteins [36] is recognized by the piRNA processing machinery. The piRNA profile associated with the *pogoN1* insertion in the *kmn1* 3′UTR resembles that of *traffic jam*, *brat*, and other genes producing 3′UTR genic piRNAs in *Drosophila* [34]. However, no transposon insertions were detected in the 3′UTRs of these genes. We hypothesize that a target site for some abundant piRNA/miRNA/siRNA located within the 3′UTR of *traffic jam* and some other genes is responsible for the generation of piRNAs by their 3′UTRs.

## Materials and Methods

### Sequencing and analysis of small RNAs

Small RNAs (19–29 nt in size) from total ovarian RNA of *y; cn bw sp* strain were prepared, sequenced and analyzed as described previously [19]. Small RNA sequencing data are deposited at Gene Expression Omnibus database under GSE46105. The mapping of small RNAs to dm3 genome assembly was performed by bowtie program [37] requiring perfect matching. In total, 16.3 million of reads were obtained and 66% of them were mapped to the genome. The small RNA library of *w^K^* strain was reported earlier ([19], GSM1024091). The library of ovarian small RNAs of *w^1118^* strain was obtained from GEO, GSM919410.

### Sequencing and analysis of *w^K^* genome

Pared-end library of fragmented genomic DNA of *w^K^* strain was prepared according to the Illumina standard protocol and sequenced on the Illumina HiSeq 2000. *w^K^* genomic deep sequencing data are deposited at NCBI SRA Database (SRR831712). The assembly was performed using the dm3 reference genome and BWA-MEM program [38] with default settings (up to 3 mismatches allowed). In total, 19.7 million of 2×100 bp reads (39.4 mio) with ∼30× coverage in euchromatin regions were obtained. Identification of deletions in the *w^K^* genome was carried out with DELLY [20]; only deletions within the range of 0.18–10 kb and at least 10× read coverage (mapQ≥15) confirmation were taken into account. Insertions of TEs in *w^K^* were identified as previously described [21] with the repeat-masked dm3 reference genome and BWA-MEM program.

### Identification of TEs that produce piRNAs in the flanking sequences

All TEs annotated for the *y; cn bw sp* strain were retrieved from the UCSC Genome Browser databases [39]. After filtering out the heterochromatic chromosome regions (chr2LHet, chr2RHet, chr3LHet, chr3RHet, chrXHet, chrYHet, chr4, chrU and chrUextra), known piRNA master-loci [6] and nested TEs, 6248 damaged and full-length copies of euchromatic individual TEs remained. For the analysis, we selected 3085 TE copies of at least 180 nt in length. TE was considered full-length if its length was at least 90% of the canonical TE; otherwise it was classified as a partial sequence. The estimation of TE sequence divergence was based on the UCSC annotation data [39]. The calculation of small RNAs originating from the surrounding regions of the TEs was performed with uniquely mapped small RNAs (reads corresponding to rRNA, tRNA, miRNA, sn/snoRNA, satellite and TE sequences were eliminated). Sequences flanking TEs were named as left and right according to the TE location in genome, ignoring the orientation of the TE itself. The statistical significance of the observed differences between amounts of small RNAs mapped to the opposite strands in sequences flanking TEs was estimated by the two-sided Student's t-test.

### In situ hybridization with polytene chromosomes

Fluorescence *in situ* hybridization (FISH) with polytene chromosomes of *y; cn bw sp* was performed as described previously [40]. The *I*-element probe contained a fragment corresponding to 745–1578 nucleotides of GenBank, acc. number M14954. The probe was labeled using the Bionick labeling system (GibcoBRL, Life Technologies).

### Inverse PCR to detect newly transposed TEs

Genomic DNA of *y; cn bw sp* strain (∼0.5 µg) was digested using the *RsaI* restriction enzyme. After inactivation of the enzyme, restriction fragments were ligated overnight at 4°C in a 200 µl volume in the presence of 1 U of T4 ligase (Promega) and precipitated. For primers used in the inverse PCR see Table S7. PCR fragments were purified with a PCR purification kit (Qiagen) and sequenced.

## Supporting Information

Figure S1 Analysis of small RNAs overlapping the borders between full-length TEs and their adjacent genomic sequences in the genome of *y; cn bw sp*. (A) The amounts of border piRNAs specific for *y; cn bw sp* strain full-length TEs mapped to the opposite strands. (B) Length distribution of border small RNAs and percentages of reads having 1 U are indicated for each strand. (C) Small RNAs overlapping the border between *412* and *F*-element TEs and neighboring genomic sequence in *y; cn bw sp* are represented. Genomic sequences are shown in upper case, TE sequences in lower case. The number of reads and their lengths are indicated.
(PDF)

Figure S2 Insertion of the *blood* retrotransposon in the intergenic region induces divergent transcription. (A) Scheme of TE insertion (chr2L:20303216–20310626) and primers used in RT-PCR. (B) Strand-specific RT-PCR reveals transcription from the *blood* into the adjacent genomic region in *y; cn bw sp*. Reverse transcription was done using primers L_RT and R_RT for left and right adjacent regions, respectively. Primers used for PCR are indicated on top. Primers L_S/L_LTR detect transcripts corresponding to the bottom genomic strand (−); R_LTR/R_AS detect RNAs corresponding to the top genomic strand (+). (C) RT-qPCR analysis of the transcription level in the *blood* flanking regions in ovaries of *y; cn bw sp* and *w^K^*. Reverse transcription was done using primers L_RT and R_RT for left and right flanks, respectively. Primers used for PCR are shown above the bars. Primers L_S/L_AS detect transcripts corresponding to the bottom genomic strand (−); R_S/R_AS detect RNAs corresponding to the top genomic strand (+).
(PDF)

Figure S3 Asymmetry in small RNA production by TE-flanking regions for copies common to *y; cn bw sp* and *w^K^* strains. (A) The distribution and amount of small RNAs produced by the opposite strands of the regions flanking full-length TEs common to *y; cn bw sp* and *w^K^*. (B) The same analysis was done for the full-length TEs common to both strains that have at least five small RNA reads within the 1-kb flanking regions from both sides of the TE insertions. *P-value* was evaluated by one-sided Student's t-test.
(PDF)

Figure S4 Ping-pong pairs are formed by heterogeneous small RNAs (related to Figure 2). Ping-pong pairs found within regions 1,2,3,4 indicated in Figure 2 are shown. The number of reads and their lengths are indicated.
(PDF)

Table S1 Full-length and partial TEs annotated in *y; cn bw s*p genome and absent from *w^K^* genome having ≥5 small RNA reads in each of their flanking regions.
(XLS)

Table S2 TE insertions revealed in *w^K^* genome and absent from the *y; cn bw sp* genome associated with *w^K^* specific small RNAs.
(XLS)

Table S3 Full-length TE insertions common to *y; cn bw sp* and *w^K^* strains. TEs that have at least five small RNA reads within the 1-kb flanking regions from both sides of the TE insertions and represented in Figure S3B are indicated by yellow.
(XLS)

Table S4 Prediction of the non-annotated TE insertions in the genome of the *y; cn bw sp* strain based on the presence of diverged piRNA clusters. The genomic coordinates of the piRNA clusters and results of inverse PCR are represented.
(XLS)

Table S5 Comparison of *I*-element insertion sites in the annotated genome and in Moscow *y; cn bw sp* sub-line.
(XLS)

Table S6 *pogoN1* insertions revealed in *y; cn bw sp* genome. Since applied deletion search procedure [20] is less effective in identification of small deletions (<200 nt), the absence of several *pogoN1* insertions in *w^K^* strain was confirmed only by PCR of genomic DNA.
(XLS)

Table S7 Primers used in this study (5′-to-3′).
(XLS)
