# Supplementary material for: Euchromatic Transposon Insertions Trigger Production of Novel Pi- and Endo-siRNAs at the Target Sites in the Drosophila Germline
Source: PLoS Genet. 2014 Feb 6;10(2):e1004138. doi: 10.1371/journal.pgen.1004138 (PMC3916259; doi:10.1371/journal.pgen.1004138)
Supplement: Figure S1 — Analysis of small RNAs overlapping the borders between full-length TEs and their adjacent genomic sequences in the genome of y; cn bw sp. (A) The amounts of border piRNAs specific for y; cn bw sp strain full-length TEs mapped to the opposite strands. (B) Length distribution of border small RNAs and percentages of reads having 1 U are indicated for each strand. (C) Small RNAs overlapping the border between 412 and F-element TEs and neighboring genomic sequence in y; cn bw sp are represented. Genomic sequences are shown in upper case, TE sequences in lower case. The number of reads and their lengths are indicated. (PDF) [file pgen.1004138.s001.pdf]

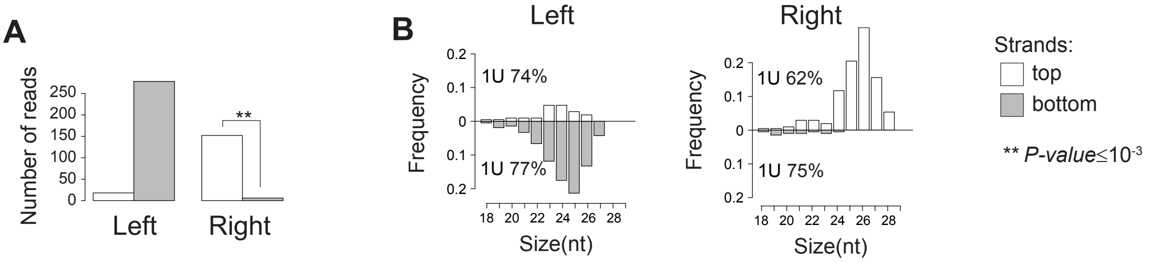

C

412, Left border
[chr2R:19417125..19417165]

```
(+)ACTTGGCTGCCTGCTCGGTGCGTCCCTtgtaatgatgaact
(-)TGAACCGACGGACGAGCCACGCAGGGAacattactacttga     Size  Reads

(-)__AACCGACGGACGAGCCACGCAGGGAac____________     27    4
(-)___ACCGACGGACGAGCCACGCAGGGAac____________     26    1
(-)____CCGACGGACGAGCCACGCAGGGAac____________     25    3
(-)_____CGACGGACGAGCCACGCAGGGAac____________     24    5
(-)______GACGGACGAGCCACGCAGGGAac____________     23    2
(-)________CGGACGAGCCACGCAGGGAac____________     21    2
(-)_________GGACGAGCCACGCAGGGAac____________     20    2
(-)___ACCGACGGACGAGCCACGCAGGGAaca___________     27    1
(-)____CCGACGGACGAGCCACGCAGGGAaca___________     26    4
(-)_____CGACGGACGAGCCACGCAGGGAaca___________     25    18
(-)______GACGGACGAGCCACGCAGGGAaca___________     24    2
(-)_______ACGGACGAGCCACGCAGGGAaca___________     23    1
(-)_________GGACGAGCCACGCAGGGAaca___________     21    2
(-)____CCGACGGACGAGCCACGCAGGGAacat__________     27    6
(-)_____CGACGGACGAGCCACGCAGGGAacat__________     26    59
(-)______GACGGACGAGCCACGCAGGGAacat__________     25    8
(-)_______ACGGACGAGCCACGCAGGGAacat__________     24    1
(-)________CGGACGAGCCACGCAGGGAacat__________     23    6
(-)_________GGACGAGCCACGCAGGGAacat__________     22    3
(-)____________CGAGCCACGCAGGGAacat__________     19    2
(-)________CGGACGAGCCACGCAGGGAacattact______     27    1
(-)________________CCACGCAGGGAacattactact___     22    1
```

412, Right border
[chr2R:19424660..19424695]

```
(+)cacatactacaCCCTGGTAGACGATCCTGCCGC
(-)gtgtatgatgtGGGACCATCTGCTAGGACGGCG    Size  Reads

(+)__catactacaCCCTGGTAGACGATC_______    24    1
(+)____tactacaCCCTGGTAGACGATCCT_____    24    2
(+)____tactacaCCCTGGTAGACGATCCTG____    25    1
(+)____tactacaCCCTGGTAGACGATCCTGC___    26    5
(+)____tactacaCCCTGGTAGACGATCCTGCC__    27    3
```

F-element, Left border
[chr3L:14260521..14260553:+]

```
(+)TATCTGCACTccttctctaccaacaaatcaagaaag
(-)ATAGACGTGAggaagagatggttgtttagttctttc    Size  Reads

(-)__AGACGTGAggaagagatggttgt___________    23    1
(-)___GACGTGAggaagagatggttgtttagt______    27    1
(-)____ACGTGAggaagagatggttgtttagt______    26    6
(-)_____CGTGAggaagagatggttgtttagt______    25    8
(-)______GTGAggaagagatggttgtttagt______    24    4
(-)_______TGAggaagagatggttgtttagt______    23    6
(-)________GAggaagagatggttgtttagt______    22    2
```
