# Supplementary figures and images for: Euchromatic Transposon Insertions Trigger Production of Novel Pi- and Endo-siRNAs at the Target Sites in the Drosophila Germline
Source: PLoS Genet. 2014 Feb 6;10(2):e1004138. doi: 10.1371/journal.pgen.1004138 (PMC3916259; doi:10.1371/journal.pgen.1004138)

**A**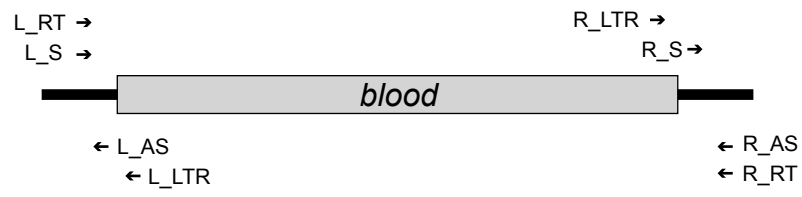**B**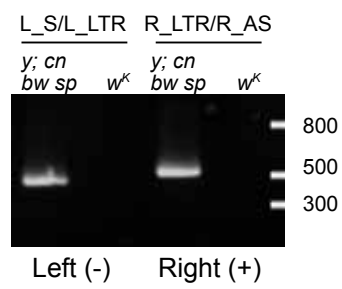**C**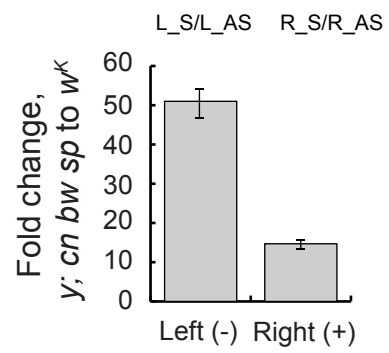

Supplement: Figure S2 — Insertion of the blood retrotransposon in the intergenic region induces divergent transcription. (A) Scheme of TE insertion (chr2L:20303216–20310626) and primers used in RT-PCR. (B) Strand-specific RT-PCR reveals transcription from the blood into the adjacent genomic region in y; cn bw sp. Reverse transcription was done using primers L_RT and R_RT for left and right adjacent regions, respectively. Primers used for PCR are indicated on top. Primers L_S/L_LTR detect transcripts corresponding to the bottom genomic strand (−); R_LTR/R_AS detect RNAs corresponding to the top genomic strand (+). (C) RT-qPCR analysis of the transcription level in the blood flanking regions in ovaries of y; cn bw sp and wK. Reverse transcription was done using primers L_RT and R_RT for left and right flanks, respectively. Primers used for PCR are shown above the bars. Primers L_S/L_AS detect transcripts corresponding to the bottom genomic strand (−); R_S/R_AS detect RNAs corresponding to the top genomic strand (+). (PDF) [file pgen.1004138.s002.pdf]

**A**

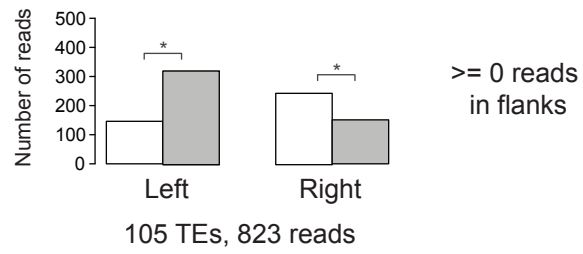

**B**

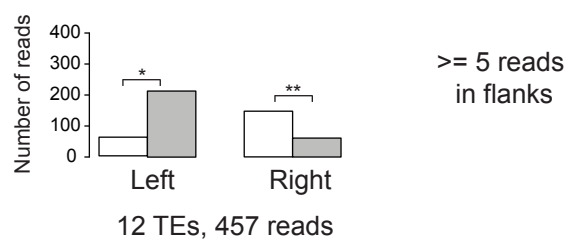

Strands: ☐ top ☐ bottom

*P-value:* \*  $\leq 0.05$ , \*\*  $< 0.01$

Supplement: Figure S3 — Asymmetry in small RNA production by TE-flanking regions for copies common to y; cn bw sp and wK strains. (A) The distribution and amount of small RNAs produced by the opposite strands of the regions flanking full-length TEs common to y; cn bw sp and wK. (B) The same analysis was done for the full-length TEs common to both strains that have at least five small RNA reads within the 1-kb flanking regions from both sides of the TE insertions. P-value was evaluated by one-sided Student's t-test. (PDF) [file pgen.1004138.s003.pdf]
