# Supplementary material for: Euchromatic Transposon Insertions Trigger Production of Novel Pi- and Endo-siRNAs at the Target Sites in the Drosophila Germline
Source: PLoS Genet. 2014 Feb 6;10(2):e1004138. doi: 10.1371/journal.pgen.1004138 (PMC3916259; doi:10.1371/journal.pgen.1004138)
Supplement: Figure S4 — Ping-pong pairs are formed by heterogeneous small RNAs (related to Figure 2). Ping-pong pairs found within regions 1,2,3,4 indicated in Figure 2 are shown. The number of reads and their lengths are indicated. (PDF) [file pgen.1004138.s004.pdf]

## Region 1
[chr2R:20650849..20650938]

```
(+) CGCGGCCGCCGAGCAGGAAGATCCAGACCCAGACCAGGAGCAGGACCGAGGCCGGGAATCGCCCATTGGTTCGTTGGAGTGCGCCCCC
(-) GCGCCGGCGGCTCGTCCTTCTAGGTCTGGGTCTGGTCCTCGTCCTGGCTCCGGCCCTTAGCGGGTAACCAAGCAACCTCACGCGGGGG    Size  Reads

(+) _________________AAGATCCAGACCCAGACC_____________________________________________________    18    1
(-) _CGCCGGCGGCTCGTCCTTCTAGGTCT_____________________________________________________________    26    5
(-) ___CCGGCGGCTCGTCCTTCTAGGTCT_____________________________________________________________    24    2
(-) _____GGCGGCTCGTCCTTCTAGGTCT_____________________________________________________________    22    1
(-) _______CGGCTCGTCCTTCTAGGTCT_____________________________________________________________    20    1
(+) __________________________________________________________TCGCCCATTGGTTCGTTGGAGTGCGCC___    27    1
(-) ___________________________________________CTGGCTCCGGCCCTTAGCGGGTAAC____________________    25    1
(+) _________________________________________________________________TTGGTTCGTTGGAGTGCGCCC__    21    1
(-) _________________________________________________CCGGCCCTTAGCGGGTAACCAAGCAA_____________    26    2
(-) __________________________________________________CGGCCCTTAGCGGGTAACCAAGCAA_____________    25    1
(-) ___________________________________________________GGCCCTTAGCGGGTAACCAAGCAA_____________    24    1
(+) __________________________________________________________________TGGTTCGTTGGAGTGCGCCC__    20    1
(-) __________________________________________________CGGCCCTTAGCGGGTAACCAAGCAAC____________    26    2
```

## Region 2
[chr2R:20653384..20653434]

```
(+) CAATGACTCAGACATCCTGCTCACGGACAAGGGCTCCCGCATCGGCAT
(-) GTTACTGAGTCTGTAGGACGAGTGCCTGTTCCCGAGGGCGTAGCCGTA    Size  Reads

(+) __________GACATCCTGCTCACGGACAAGGGC______________    24    1
(+) __________GACATCCTGCTCACGGACAAGGGCTCC___________    27    1
(-) __TACTGAGTCTGTAGGACG____________________________    18    1
(+) _________________TGCTCACGGACAAGGGCTCCCG_________    22    1
(+) _________________TGCTCACGGACAAGGGCTCCCGCA_______    24    1
(+) _________________TGCTCACGGACAAGGGCTCCCGCATC_____    26    5
(-) _TTACTGAGTCTGTAGGACGAGTGCCT_____________________    26    2
(-) __TACTGAGTCTGTAGGACGAGTGCCT_____________________    25    1
(+) ____________________TCACGGACAAGGGCTCCCG_________    19    1
(+) ____________________TCACGGACAAGGGCTCCCGCATCGG___    25    2
(+) ____________________TCACGGACAAGGGCTCCCGCATCGGC__    26    2
(-) ____CTGAGTCTGTAGGACGAGTGCCTGTT__________________    26    1
(+) _____________________CACGGACAAGGGCTCCCGCAT______    21    1
(+) _____________________CACGGACAAGGGCTCCCGCATCGGC__    25    2
(-) __________CTGTAGGACGAGTGCCTGTTC_________________    21    1
```

## Region 3
[chr3L:21203682..21203747]

```
(+) GCGAATGCGACTTTAAACGTCCATAAACATTCAGGCAGAAAACGAAAATAATAAAACGACGGTCGTTCGG
(-) CGCTTACGCTGAAATTTGCAGGTATTTGTAAGTCCGTCTTTTGCTTTTATTATTTTGCTGCCAGCAAGCC    Size  Reads

(+) _________________CGTCCATAAACATTCAGGCAG________________________________    21    3
(-) ___TTACGCTGAAATTTGCAGGTATTT_GGGGGGGGG__________________________________    24    1
(+) ______________________________________AAAACGAAAATAATAAAACGACGGTC______    26    1
(-) ___________________________GTAAGTCCGTCTTTTGCTTTT______________________    21    1
(+) ___________________________________________GAAAATAATAAAACGACGGTC______    21    1
(-) __________________________TGTAAGTCCGTCTTTTGCTTTTATTAT_________________    27    1
(-) ___________________________GTAAGTCCGTCTTTTGCTTTTATTAT_________________    26    1
(-) __________________________________CGTCTTTTGCTTTTATTAT_________________    19    1
```

## Region 4
[chr3L:21204145..21204199]

```
(+) GGGGGCGTAAGTCGCTTACTTTAAAATGATTGGGTGGTAGGCGTCCGGTT
(-) CCCCCGCATTCAGCGAATGAAATTTTACTAACCCACCATCCGCAGGCCAA    Size  Reads

(+) ________________TACTTTAAAATGATTGGGTGG_____________    21    2
(+) ________________TACTTTAAAATGATTGGGTGGTA___________    23    1
(+) ________________TACTTTAAAATGATTGGGTGGTAG__________    24    2
(+) ________________TACTTTAAAATGATTGGGTGGTAGGC________    26    1
(-) _CCCCGCATTCAGCGAATGAAATTTT________________________    25    1
(+) _____________________TAAAATGATTGGGTGGTAGG_________    20    2
(+) _____________________TAAAATGATTGGGTGGTAGGCGTCCG___    26    1
(+) _____________________TAAAATGATTGGGTGGTAGGCGTCCGG__    27    1
(-) __________CAGCGAATGAAATTTTACTAA___________________    21    1
```
